# Culturally Informed Dietary Approaches for Cardiometabolic Risk Reduction in South Asians: An Evidence-Based Review

**DOI:** 10.3390/jcm15041421

**Published:** 2026-02-11

**Authors:** Ananya Pappu, Nishita Mishra, Sneha Mishra

**Affiliations:** 1 Mayo Clinic Alix School of Medicine, Mayo Clinic Arizona, 5951 E. Mayo Blvd., Phoenix, AZ 85054, USA; 2 BASIS Scottsdale, Basis Charter Schools, 10400 N. 128th St., Scottsdale, AZ 85259, USA; mishrnishita@gmail.com; 3 Division of Community Internal Medicine, Mayo Clinic Arizona, 13400 E. Shea Blvd., Scottsdale, AZ 85259, USA; mishra.sneha@mayo.edu

**Keywords:** cardiometabolic risk, cardiovascular disease, dietary patterns, primary care interventions, South Asians, whole-food plant-based diet

## Abstract

**Background/Objectives:** South Asians (SAs), including individuals from India, Pakistan, Bangladesh, and Sri Lanka, experience a disproportionately high burden of cardiometabolic disease at lower body mass index (BMI) thresholds than other populations. Rates of cardiovascular disease and diabetes are elevated even below the standard overweight BMI range defined by the World Health Organization. Because dietary practices are strongly influenced by cultural and religious traditions, culturally tailored interventions are essential for effective cardiometabolic risk reduction. This article serves as an initial approach to dietary guidance in the SA community. **Methods:** A literature review was conducted using the Ovid MEDLINE(R) ALL database to identify English-language studies evaluating dietary interventions in SA populations. Studies were selected through title and abstract screening, with additional articles identified through reference review. **Results:** Several dietary strategies were associated with improved cardiometabolic outcomes in SA. Diets emphasizing whole, minimally processed plant-based foods and reduced refined carbohydrate intake were associated with improvements in weight, BMI, lipid profiles, and glycemic control. Recommended dietary patterns included non-starchy vegetables, plant-based proteins such as lentils, and whole grains. Intermittent fasting approaches, including time-restricted eating and the 5:2 diet, demonstrated benefits for insulin sensitivity and aligned with traditional fasting practices common in SA cultures. **Discussion:** Future studies are needed to better characterize the dietary diversity within the SA community. Public health initiatives should focus on education and early intervention. **Conclusions:** Dietary interventions aligned with cultural South Asian food practices may improve adherence and reduce cardiometabolic risk.

## 1. Introduction

The South Asian (SA) population comprises people with ancestry from the 8 countries of India, Pakistan, Bangladesh, Sri Lanka, Nepal, Bhutan, the Maldives, and Afghanistan [1]. As of 2024, this population surpassed 2 billion people [2]. In addition, Asian Americans are the fastest-growing group in the US [3], with Indian Americans constituting the second-largest Asian-origin group in the US, with 4.6 million people [4].

South Asians, both in their native countries [5] and countries abroad such as the US [6] and the UK [7], have higher rates of atherosclerotic cardiovascular disease than other populations. This observation is multifactorial and influenced by several determinants, including genetics, socioeconomic status, and cultural norms surrounding healthy behaviors [8]. For example, central adiposity and insulin resistance are common among South Asians, even those with a lower BMI (body mass index, calculated as weight in kilograms divided by height in meters squared), and contribute significantly to the increased incidence of cardiovascular disease in this population [9]. Whereas a BMI of 25 to 29.9 is considered overweight for non-Asians, the overweight range for Asians is 23 to 27.4 due to different associations between BMI and health risks. Furthermore, the combination of low physical activity levels with dietary patterns rich in refined carbohydrates and saturated fats [10] exacerbates cardiometabolic risk in the SA population.

In light of the rapidly expanding SA population worldwide, culturally tailored medical interventions and public health strategies are urgently needed. This need can be addressed by several approaches that optimize the unique dietary patterns of South Asians. Here, we aim to provide evidence-based dietary recommendations that are culturally sensitive and designed to meet the needs of the SA diaspora.

## 2. Materials and Methods

A comprehensive literature search was conducted using the Ovid MEDLINE(R) ALL database to identify studies evaluating dietary interventions in South Asian populations with diabetes mellitus or cardiovascular disease. Searches were limited to English-language articles published during the 21st century (1 January 2001 through the date of the search, 10 July 2024). The search strategy incorporated both Medical Subject Headings (MeSH) and keyword terms to ensure broad capture of the relevant literature. Specifically, the following concepts were combined using Boolean operators:

1. Dietary interventions
  - MeSH and keyword terms: “Diet Therapy”[MeSH] OR “diet* intervention*”.
2. Chronic disease outcomes of interest
  - MeSH terms: “diabetes mellitus”[MeSH] OR “cardiovascular diseases”[MeSH].
3. South Asian populations
  - Keywords and MeSH terms: “south* asia” OR “Asia, Southern”[MeSH].

The combination of these three concepts using AND yielded 131 articles in English for initial screening.

All retrieved records were reviewed for relevance to diet-based interventions or dietary patterns in South Asian populations in relation to diabetes mellitus or cardiovascular disease outcomes. Titles and abstracts were screened initially, with full-text review performed when eligibility was unclear. Studies were eligible for inclusion if they examined South Asian populations residing either on the South Asian subcontinent or within South Asian diaspora communities, focused on adult participants aged 18 years or older, and evaluated dietary interventions, dietary patterns, or culturally relevant nutritional exposures with respect to metabolic or cardiovascular outcomes. Both interventional and observational study designs, as well as systematic and narrative reviews, were considered eligible when aligned with these criteria.

Articles were excluded if they did not align with the population, intervention, or outcomes of interest. Specifically, studies were excluded if they were conducted in non-South Asian populations (*n* = 1) or focused exclusively on pediatric populations (*n* = 5); evaluated outcomes not directly related to cardiometabolic health, such as grip strength (*n* = 1); centered on rare genetic or cholestatic conditions unrelated to dietary cardiometabolic risk (e.g., Alagille syndrome; *n* = 1); or primarily examined environmental or toxic exposures without a dietary intervention focus (e.g., metal exposure; *n* = 1). Additional exclusions included studies limited to South Asian patients with celiac disease, in which dietary exposure reflected disease-specific restriction rather than broader dietary patterns (*n* = 5), as well as studies focused on pregnancy or menopause without relevance to cardiovascular or metabolic outcomes (*n* = 2). One duplicate record was also removed. In total, 17 articles were excluded, yielding 114 articles for further review.

Additional sources were identified using a snowball approach, which included reviewing references of included studies and conducting targeted follow-up searches based on themes that were underrepresented in the initial search. For example, if a dietary pattern (such as vegetarian diets, low-glycemic South Asian staple modifications, or culturally adapted heart-healthy diets) appeared frequently but lacked mechanistic or comparative outcome data, supplementary searches were conducted using narrower, diet-specific terms. This iterative process continued until additional searches no longer contributed significantly to the overall understanding of dietary interventions relevant to South Asian metabolic and cardiovascular health.

All articles identified through both the primary search and snowballing process were cataloged and evaluated for study design, population characteristics, intervention components, and clinical outcomes.

## 3. Results

### 3.1. Overview of Cultural and Dietary Practices in South Asia

The diverse SA population has rich dietary practices influenced by culture, religion, and regional geography [11]. According to many Dharmic faiths, including Jainism, Buddhism, Sikhism, and Hinduism, a plant-based diet is recommended. Notably, approximately 44% of Hindus consider themselves vegetarians [12]. In practice, the vegetarian diet encompasses a broad spectrum of diets, ranging from those that exclude all animal products and emphasize non-root vegetables to those that include all plant products and also certain animal products, such as dairy or eggs (Table 1). Many South Asians are semi-vegetarian and consume animal products only on certain days of the week. Nonetheless, many Hindus who consume animal products avoid beef and pork. People of other faiths who consume meat emphasize mutton, beef, and poultry products. This dietary pattern reflects the religious practices of Islam, which stress the consumption of halal meats as an integral part of the diet [13].

Despite its vast cultural and regional diversity, the SA diet has unifying features such as a strong foundation in plant-based foods and carbohydrates, particularly those in the form of rice, lentils, beans, and flatbread. These staples are common across the subcontinent and provide a base for meals often complemented by a variety of vegetables, spices, and—depending on religious and cultural practices—proteins, in the form of dairy, poultry, fish, or meat. Regardless of the specific regional or religious variations, a plant-centric approach, with an emphasis on vegetables, legumes, and grains, can create a diet that is nutritionally dense with a balance of macronutrients and micronutrients [14].

Another unifying feature of the SA diet is the widespread use of spices and herbs to flavor food, giving SA cuisine its distinctive and vibrant flavors. The frequent use of fresh and dried spices, such as turmeric, cumin, cardamom, and coriander, is a hallmark of SA cuisine. These spices are valued not only for their flavor but also for their medicinal properties. As one interviewee of the study by Mukherjea et al. [15] stated, “All spices have some medicinal value.” This has been supported in the literature, which has shown that spices such as turmeric and cumin have anti-inflammatory properties [16] and aid with digestion [17], respectively.

The SA diet is also deeply influenced by geography, with regional variations reflecting local resources and traditions. In coastal cities, seafood such as fish is a staple [11], whereas coconut-based dishes are prevalent in tropical areas where coconuts are abundant [18]. These geographical factors shape dietary practices and have resulted in diverse and region-specific culinary traditions across South Asia.

### 3.2. Role of Food in SA Identity and Health

In SA culture, food is viewed not only as a means for sustenance and nutrition but also as a vehicle for cultural expression and social identity. Studies have shown that culinary practices in the immigrant community function as ways to participate in rituals, demonstrate faith, foster group belonging, and fulfill social roles [19]. These practices are deeply rooted in beliefs regarding the spiritual and health benefits of these foods.

Unfortunately, many of these dietary practices do not align with those that are recommended because SA diets often lack certain food groups and overemphasize others. For example, many traditional foods are rich in refined carbohydrates, most likely due to their availability and affordability, which makes their consumption convenient. The STARCH (Study To Assess the dietaRy CarboHydrate content of an Indian population with type 2 diabetes) revealed that carbohydrates constitute 64.1% of the total energy in the diet of Indian patients with type 2 diabetes [20]. However, several studies have shown that increased intake of refined grains is linked to increased risk of cardiovascular disease, cancer, and all-cause mortality, indicating that the overconsumption of starches is a dangerous choice [21].

Another key aspect of traditional SA diets is the cultural importance of sweets, which were widely discussed by participants in a study by Mukherjea et al. [15]. Many participants noted the central role of sweets in their cultural and social practices, such as marking special occasions and auspicious events. Despite health concerns about the high sugar content of traditional sweets, participants expressed a reluctance to reduce their consumption, with some even believing moderate consumption is beneficial [15].

In contrast, some traditional SA dietary practices have been shown to have beneficial health outcomes. One such practice belonging to many SA religious groups is fasting. In this context, fasting is often viewed as a sacrifice or offering to a deity and is a way to purify oneself [15]. As discussed in later sections, fasting and time-restricted eating improve cardiometabolic outcomes and may be more easily integrated into the SA culture.

### 3.3. Health Outcomes in South Asians and Nutritional Challenges Linked to Diet

#### 3.3.1. Health Outcomes in South Asians

Initiated in 2010, the landmark MASALA (Mediators of Atherosclerosis in South Asians Living in America) study is a longitudinal community-based prospective cohort study involving patients of SA ancestry who were primarily immigrants of Indian and Pakistani origin living in the San Francisco Bay area or the greater Chicago area. For the initial study, data were collected for patient demographic characteristics, lifestyle and psychosocial factors, standard cardiovascular disease risk factors, oral glucose tolerance testing, electrocardiography, microalbuminuria assessment, coronary artery calcium measurement, and abdominal visceral fat measurement obtained by using computed tomography [22]. The South Asians of the MASALA study group had a significantly higher age-adjusted prevalence of diabetes (23%) than the African American, Chinese American, Latino, or White participants of the MESA (Multiethnic Study of Atherosclerosis) ethnic groups (18% in African Americans, 13% in Chinese Americans, 17% in Latinos, and 6% in Whites) [23,24]. Furthermore, the MASALA study showed that South Asians had adverse body composition in addition to lower insulin secretion, more insulin resistance, and higher coronary artery calcium density than other US racial and ethnic groups [25,26].

#### 3.3.2. BMI in South Asians

Traditionally, assessing the risk of cardiovascular disease involves using BMI as a metric to identify people who are overweight and obese, especially in Western populations. For non-Asians, a BMI of 25 to 30 is classified as pre-obese, whereas a BMI greater than 30 is considered obese [27]. However, for Asians, BMI ranges are lower because the prevalence of diabetes and cardiovascular disease is increased among Asians at relatively lower BMIs than other populations. This is in part due to the observation that all Asians, including Indians, have a high percentage of body fat at low BMIs [28], resulting in an increased risk of disease. The World Health Organization recommends the following BMI ranges for classifying South Asians at risk for cardiovascular disease: less than 18.5, underweight; 18.5 to 22.9, increasing but acceptable risk; 23 to 27.4, increased risk; and 27.5 or greater, high risk [27,29]. Given the underlying predisposition of South Asians to developing cardiometabolic disease at lower BMIs, adopting culturally specific dietary guidelines and interventions for the early screening and management of at-risk Asian populations is imperative.

#### 3.3.3. Micronutrient Deficiencies

Research has shown that South Asians are at increased risk for several micronutrient deficiencies. The widespread practice of vegetarianism in this population frequently leads to deficiencies in vitamin B12, a micronutrient obtained from animal products [30]. Although vegetarians are at increased risk for vitamin B12 deficiency, population studies have revealed this deficiency in both vegetarians and non-vegetarians [31]. Vitamin B12 deficiency is associated with hematologic disorders such as anemia, peripheral neuropathy, and neuropsychiatric impairments [32,33].

Vitamin D (25-hydroxyvitamin D) deficiency is also a pervasive health issue in all SA countries [34], as well as in Western countries, including Canada and the UK [35]. People with vitamin D deficiency are most notably at risk for osteomalacia and osteoporosis [36].

#### 3.3.4. Macronutrient Deficiencies

Protein deficiency is a common macronutrient deficiency among South Asians. Of the various SA diets, vegetarianism may carry the highest risk of protein deficiency because it excludes protein from many—if not all—animal sources. Although South Asians living in America consume more protein than those living in South Asia, results of a systematic review suggested that they may consume less than other Westerners [37]. Additionally, a negative association has been observed between protein intake and length of residence for South Asians in the US, whereby increased duration of residence was associated with decreased protein intake [38].

### 3.4. Evidence-Based Recommendations for Dietary Modifications

#### 3.4.1. Whole-Food, Plant-Based Diet

**Figure 1:**
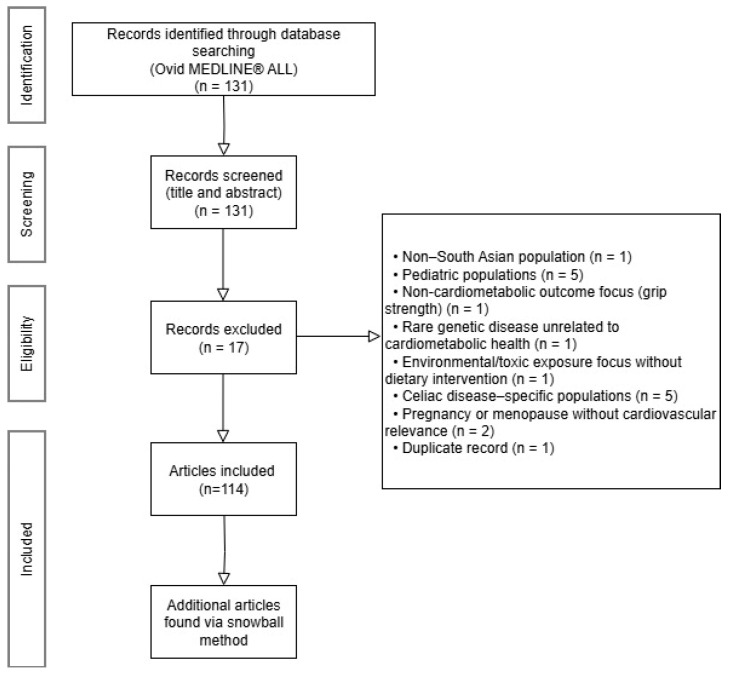
Flow diagram of studies considered for inclusion in the review.

Considering the unique challenges faced by South Asians, optimizing lifestyle management for cardiometabolic risk factors is crucial. Adopting a whole-food, plant-based (WFPB) diet (Figure 1) [39] is one strategy to reduce these risks. A WFPB diet has been shown to decrease cardiometabolic risk by lowering weight, BMI, total cholesterol levels, hemoglobin A1c levels, and the risk of metabolic dysfunction-associated steatotic liver disease [40].

Compared with the US Department of Agriculture’s nutrition guide, MyPlate, the WFPB diet recommends more total vegetables (180%), green leafy vegetables (238%), legumes (460%), whole fruit (100%), and whole grains (132%) and less refined grains (−74%) [14]. More specifically, the WFPB diet suggests that one-half of a meal should consist of non-starchy vegetables. In the SA diet, this would include traditional vegetables such as okra, tindora, and eggplant and leafy greens such as amaranth, collards, chard, and mustard. With the WFPB diet, one-quarter of the meal should include plant-based protein, such as the SA staples of cooked beans, lentils, and paneer, and the remaining quarter of the meal should comprise healthy grains or starches, such as whole-wheat bread, millets, tapioca, and barley. Given the large number of vegetarians in the SA community, the WFPB diet aligns well with existing cultural norms.

In the BROAD study conducted in New Zealand, participants were randomly assigned to a group that consumed the WFPB diet or to a control group with no intervention. Follow-up at 6 and 12 months showed that the mean BMI of the WFPB diet group was significantly reduced by 4.4 (range, 0.4–7.4; 95% CI, 3.7–5.1) and 4.2 (range, 0.5–8.3; 95% CI, 3.4–5), respectively [40]. It is important to note that BMI alone does not distinguish between changes in fat mass and lean mass, and reductions in BMI may partly reflect changes in muscle rather than isolated reductions in adiposity. Despite these limitations, the observed reductions in BMI suggest potential metabolic benefit. Given the success of the WFPB diet in White populations, trials of its adoption in SA communities are warranted.

#### 3.4.2. Nutritional Considerations and Supplementation

It is important to note that individuals consuming a strict plant-based diet may be at risk for certain micronutrient deficiencies. Whole-food, plant-based dietary patterns do not consistently meet the Recommended Dietary Allowances (RDA) for vitamin B12 and vitamin D, and may also fall short of calcium requirements in certain populations, particularly women aged 51–70 years [14]. These nutrients are more abundantly or exclusively obtained from animal-derived foods, underscoring the importance of appropriate supplementation when adopting a strict plant-based diet.

For individuals following a vegan diet, vitamin B12 supplementation is essential, with commonly recommended dosing ranging from 50 to 100 µg daily, although higher intermittent dosing regimens up to 2000 µg may also be used [41]. As vitamin B12 is water-soluble, no adverse effects of oversupplementation have been reported [41]. Vitamin D supplementation is also recommended, with typical dosing of 1000–2000 IU (25–50 µg) daily, depending on baseline status and individual risk factors [42]. Calcium supplementation is recommended as 1000 mg for men and women aged 19–50 years, and 1200 mg for women over 51 and men over 71 [43]. 

**Figure 2:**
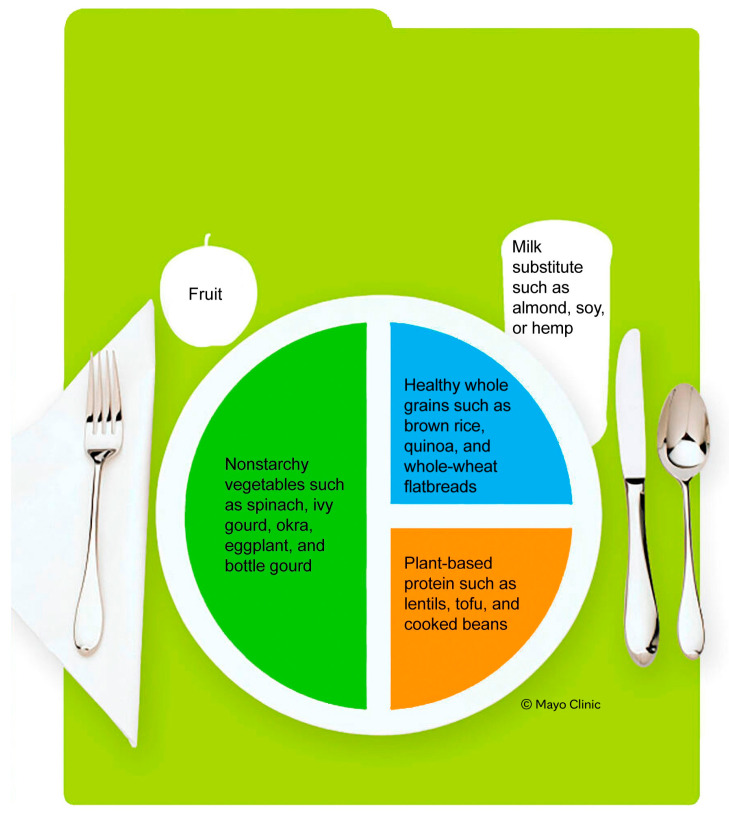
The Plant-Based Plate [39]. (Modified from Mayo Clinic. Patient Education: Diabetes Self-Management: Diabetes Overview. Mayo Foundation for Medical Education and Research. 2016; used with permission of Mayo Foundation for Medical Education and Research).

Additional nutrients that warrant consideration include iron, given reduced bioavailability of non-heme iron from plant sources; omega-3 fatty acids, particularly EPA and DHA, which may be limited in the absence of fish or algae-based sources; and iodine, especially among individuals who avoid iodized salt or dairy products [44]. Counseling on supplementation and dietary sources should be incorporated into clinical guidance to ensure nutritional adequacy and long-term safety (Figure 2).

#### 3.4.3. Reducing Simple Carbohydrate Intake

Research findings indicate that reducing the intake of simple carbohydrates can result in weight loss and an improved insulin response. In a study of 23 overweight, insulin-resistant women of SA ancestry living in California, participants were instructed to begin a diet consisting of 40% to 45% carbohydrates, equating to about a 500 kcal caloric deficit per day. The women were provided with a 1 to 2 h educational session regarding this new diet and food substitutions for items in the traditional SA diet. For instance, participants were advised to reduce carbohydrate portions, use brown rice and cracked wheat in place of white rice, and incorporate tofu and ground nuts into the traditional atta used for making rotis and chapatis. Over a 3-month time frame, 19 of the 23 women had lost more than 5 lb and had a significantly reduced mean (SD) BMI (30.2 [0.8] vs. 28.2 [0.8]; *p* < 0.0001) [45]. Furthermore, the mean (SD) steady-state plasma glucose concentration decreased from 217 (12) mg/dL to 176 (16) mg/dL (*p* < 0.001), triglyceride levels decreased from 137 mg/dL to 101 mg/dL (*p* = 0.003), and glucose levels decreased from 98 mg/dL to 92 mg/dL (*p* = 0.005). This study suggested that insulin-resistant women with SA ancestry can lose weight and improve their insulin resistance by adopting a calorie-restricted, relatively low-carbohydrate diet [45].

#### 3.4.4. Substituting Unhealthy Fats

A key ingredient in many traditional South Asian sweets and a common source of dietary fat is clarified butter, known as ghee. Ghee is widely used in cooking and is considered an essential component of many households. It is produced by heating butter to remove water and milk solids, resulting in primarily butterfat [46]. Although ghee is traditionally valued for its flavor, culinary stability at high cooking temperatures, and cultural significance, its high saturated fat content warrants moderation. Dorni et al. analyzed the fatty acid composition of 107 ghee samples collected across India and found that ghee consists predominantly of saturated fatty acids (71.02 ± 1.86%), with smaller contributions from monounsaturated fatty acids (26.44 ± 1.63%) and polyunsaturated fatty acids (2.54 ± 0.64%). Other potentially unhealthy fats used in the SA diet include coconut oil, which in this study demonstrated the highest total saturated fatty acid content of all oils (90.84%) [47]. A study included 30 healthy men and women who were randomized to use ghee or olive oil in their cooking for 4 weeks. Compared with the olive oil, the patients who consumed a diet with ghee increased fasting plasma apo-B (0.09, 95% CI 0.02, 0.17 g/L, *p* = 0.018) and non-HDL-cholesterol (0.53, 95% CI 0.01, 1.05 mmol/L, *p* = 0.046) [48]. Excessive intake, therefore, may contribute to dyslipidemia and increased cardiometabolic risk, particularly in South Asian populations who are predisposed to insulin resistance at lower body mass index thresholds. Therefore, the National Lipid Association recommends replacing ghee with an unsaturated oil such as olive oil or corn oil [49].

#### 3.4.5. Intermittent Fasting

Another strategy to losing weight and improving the insulin response is known as intermittent fasting, which encompasses time-restricted eating, alternate-day fasting (ADF), and the 5:2 diet [50]. This dietary strategy involves limiting caloric intake to specific time frames, which can benefit metabolic health, particularly with respect to insulin sensitivity and cardiometabolic outcomes. In addition, intermittent fasting can synchronize eating patterns with the body’s circadian rhythm, which optimizes metabolic processes. For instance, early time-restricted feeding, which combines time-restricted eating (food consumed during a 6 h window) and early eating (dinner consumed before 3 PM), has been shown to improve insulin sensitivity and reduce the risk of type 2 diabetes in men who are overweight [51]. This is achieved partly through the activation of metabolic pathways that shift metabolism away from lipid synthesis, cholesterol synthesis, and fat storage and instead shift it toward the mobilization of fat, which enhances cellular efficiency and reduces oxidative stress [52,53]. The ADF diet involves alternating between days of normal calorie intake and significantly reduced or zero calorie intake. Studies have shown that, compared with common calorie-restricting practices, ADF is associated with significant weight loss [54] and improved systolic blood pressure [55]. Finally, the 5:2 diet involves regular caloric intake for 5 days followed by 2 days of fasting, with some protocols permitting up to 500 kcal per day on fasting days [56]. In a recent study, the 5:2 diet resulted in significantly greater improvement in waist circumference, fasting glucose levels, and hemoglobin A1c levels for patients with type 2 diabetes than for participants without diabetes [57]. These practices may resemble those that exist in the SA community: Islam requires fasting during the holy month of Ramadan, and people of many Dharmic faiths commonly practice fasting on particular days or weeks [58]. Therefore, recommending intermittent fasting practices to SA patients may facilitate the integration of these practices and long-term adherence.

### 3.5. Practical Tools and Resources for Primary Care Professionals

Given the cultural importance of food in the SA population, dietary approaches must be adopted to maintain traditions while promoting balanced nutritional practices. Tailored interventions that consider cultural context have been shown to promote the success of interventions in SA patients. In Table 2, common dietary challenges faced by SA patients are outlined, along with strategies that primary care physicians and other health care professionals (i.e., physician assistants, nurse practitioners, and nutritionists) may use to achieve a shared goal [59]. These recommendations aim to address sociocultural barriers and propose culturally informed recommendations.

## 4. Discussion

### 4.1. Study Limitations

Our review process is limited by several important factors. First, the predominance of short-term studies, typically ranging from 3 to 12 months, restricts conclusions regarding the long-term sustainability and durability of dietary interventions, particularly plant-based and carbohydrate-modified diets, in South Asian populations. Longer follow-up periods are needed to better assess sustained cardiometabolic outcomes and adherence over time.

Second, our literature search was conducted using a single database, Ovid MEDLINE(R), which may have resulted in the exclusion of relevant studies indexed in other databases, including regional South Asian or public health-focused repositories. Additionally, the exclusion of non-English-language studies may have led to the omission of potentially valuable research published in South Asian languages, thereby limiting representation of locally conducted interventions.

Another important limitation relates to heterogeneity across the included studies. The reviewed literature encompassed diverse study designs, dietary interventions, population characteristics, and cardiometabolic outcome measures, which limited direct comparison across studies and precluded quantitative synthesis. Moreover, commonly used outcome measures such as BMI have known limitations in South Asian populations, as cardiometabolic risk frequently manifests at lower BMI thresholds and may not fully capture central adiposity or visceral fat burden.

Finally, both within the reviewed literature and in broader public health reporting, South Asians are often grouped into a single category or aggregated with other Asian populations [60]. This practice obscures meaningful differences in dietary patterns, cultural practices, and cardiometabolic risk profiles across South Asian subgroups and regions. Disaggregating data by ethnicity, country of origin, and migration context will be essential for developing more precise and culturally responsive dietary interventions.

### 4.2. Future Directions

Future studies should focus on greater inclusion of underrepresented South Asian subgroups, including Bangladeshi, Sri Lankan, Nepali, Bhutanese, and Afghan populations. Much of the existing literature disproportionately focuses on Indian and Pakistani populations and may not adequately capture the heterogeneity of dietary practices, cultural norms, and health risks across the broader South Asian community.

Low-income South Asian communities experiencing food insecurity and economic instability remain underrepresented in dietary research, as well. Structural barriers, including limited access to affordable, healthy foods and competing socioeconomic demands, may hinder adherence to recommended dietary patterns. Studies exploring food insecurity and dietary patterns among low-income Asian American groups note heterogeneous experiences across subgroups and emphasize the need for more targeted research to understand nutritional risk among South Asians specifically [61]. Future studies should evaluate the feasibility and effectiveness of affordable, culturally appropriate dietary interventions and assess the impact of food assistance programs on cardiometabolic outcomes in these populations.

Finally, future research should incorporate more precise and population-appropriate measures of cardiometabolic risk beyond body mass index. Given the limitations of BMI in assessing adiposity and cardiometabolic risk in South Asian populations, studies should prioritize direct measures of body fat distribution, such as waist circumference, waist-to-hip ratio, or imaging-based assessments, to more accurately evaluate the impact of dietary interventions on body composition and metabolic health.

### 4.3. Public Health Implications and Policy Considerations

Addressing the dietary challenges of the SA population requires systemic public health initiatives and policy interventions. Insulin resistance and central adiposity often emerge early in life among South Asian populations; however, the dietary intervention literature, including the studies reviewed here, predominantly focuses on adults. Addressing cardiometabolic risk earlier in age represents an important public health opportunity. Preventive dietary strategies targeting South Asian children and adolescents should be prioritized and ideally implemented before the development of metabolic dysfunction and maladaptive nutritional habits. Such interventions must ensure adequate nutrient intake to support growth and development and may include school-based initiatives or family-centered counseling programs.

At the population level, education and awareness are critical components of effective prevention strategies. Educational campaigns should emphasize the cardiometabolic risks associated with excessive consumption of refined grains, sugar-sweetened beverages, and deep-fried foods, while simultaneously promoting culturally acceptable alternatives such as whole grains, legumes, and healthier cooking methods, including air-frying. Visual tools, including culturally adapted portion sizes and food pyramids, can bridge knowledge gaps [62].

Complementing educational efforts, policy-level interventions are needed to support healthier food environments and reduce structural barriers to dietary change. Policy advocacy should focus on improving the affordability and accessibility of nutrient-dense foods, including lentils, millets, and whole grains, which are central to many traditional South Asian diets. Clear nutritional labeling that highlights sodium, sugar, and fat content in South Asian packaged foods may further aid informed decision-making. In addition, community engagement through partnerships with cultural organizations can facilitate physical activity interventions [63] and cooking classes tailored to traditional recipes [64,65].

## 5. Conclusions

On average, South Asians are more likely to develop cardiovascular issues at lower BMIs or younger ages compared to other populations. This review highlights evidence-based dietary practices, including whole-food plant-based diets, carbohydrate limitations, and intermittent fasting. Beyond the physical benefits of these diets, this review emphasizes the importance of taking traditional dietary habits into consideration, which is the key to successful intervention. Recommending generic dietary advice to South Asian patients should be avoided, and focus should instead be placed on building upon the already positive aspects of traditional foods, such as legumes, spices, and plant-based staples. Simultaneously, physicians must address the common overconsumption of refined carbohydrates and fried foods common to South Asian culture. As the South Asian population continues its rapid growth worldwide, this public health challenge represents an important opportunity to demonstrate how culturally informed, evidence-based nutrition can transform population health on a global scale. Researchers, funding agencies, and healthcare systems should prioritize studies that investigate South Asian populations by ethnicity, socioeconomic status, and generational status to develop personalized nutrition approaches.

## Figures and Tables

**Table 1:** Definitions of Dietary Patterns.

Diet	Meat	Poultry	Fish	Eggs	Dairy
Vegan ^a^	No	No	No	No	No
Lactovegetarian	No	No	No	No	Yes
Lacto-ovo vegetarian	No	No	No	Yes	Yes
Pescovegetarian	No	No	Yes	Yes	Yes
Semi-vegetarian ^b^	Varies	Varies	Varies	Varies	Yes

^a^ Sometimes excludes all animal products, including honey. ^b^ Includes animal products on certain days; some religions avoid beef and pork.

**Table 2:** Culturally Sensitive Recommendations for South Asians for Addressing Key Challenges to Cardiovascular Health.

Domain	Key Challenges	Recommendations
Traditional diet	High carbohydrate content in traditional foods (e.g., rice and breads) and reliance on fried foods	Advise portion control of rice and bread and substitute with brown rice, quinoa, or whole-wheat roti. Avoid fried foods and recommend steaming or grilling. Provide culturally specific meal plans.
Physical activity	Sedentary lifestyles and limited access to culturally relevant exercise options	Encourage 30 min of walking or yoga daily. Promote culturally relevant activities such as bhangra or cricket. Suggest family-based exercises.
Language barriers	Limited English proficiency in some South Asian subgroups (e.g., Bangladeshi, Bhutanese)	Use interpreters for non-English speakers. Provide patient education materials in South Asian languages (e.g., Hindi, Bengali, Dzongkha).
Cultural sensitivity	Gender preferences for health care professionals and cultural fatalism about disease management	Offer female clinicians when requested. Educate staff on cultural values such as family involvement in decision-making and address fatalistic beliefs with motivational interviewing.
Ramadan fasting	Risk of hypoglycemia and poor meal choices during fasting	Adjust medication timing for fasting. Recommend balanced meals (e.g., complex carbs, lean proteins) at Suhoor and Iftar. Educate on hydration and glucose monitoring during fasting.
Socioeconomic barriers	Income disparities and limited access to health care among lower-resourced South Asian subgroups	Screen for food insecurity and access to medications. Refer to local low-cost clinics, community assistance programs, and patient support groups.
Technology	Low awareness of diabetes management tools tailored to South Asians	Recommend apps such as mDiab for South Asian meal tracking and diabetes education. Promote wearable activity trackers.

Data from Rahim et al. [59].

## Data Availability

No new data were created or analyzed in this study.

## Abbreviations

The following abbreviations are used in this manuscript:

ADF	Alternate-day fasting
BMI	Body mass index
MASALA	Mediators of atherosclerosis in South Asians living in America
SA	South Asian
WFPB	Whole-food, plant-based
RDA	Recommended dietary allowances
